# Interpretable Artificial Neural Network Models for Predicting Anti-Adalimumab Immune Complex and Serum Drug Level in Crohn’s Disease: A Proof-of-Concept Study

**DOI:** 10.3390/pharmaceutics17121536

**Published:** 2025-11-29

**Authors:** Livia Moreira Genaro, Juliana Carron, Gustavo Jacob Lourenço, Cristiane Kibune Nagasako, Glaucia Fernanda Soares Rupert Reis, Michel Gardere Camargo, Priscilla de Sene Portel Oliveira, Carmen Silvia Passos Lima, Maria de Lourdes Setsuko Ayrizono, Anibal Tavares de Azevedo, Raquel Franco Leal

**Affiliations:** 1 Inflammatory Bowel Disease Research Laboratory (LabDII), Gastrocenter, Colorectal Surgery Unit, Surgery Department, School of Medical Sciences, University of Campinas (Unicamp), Carlos Chagas Street, 420, Cidade Universitária Zeferino Vaz, Campinas 13083-878, SP, Brazil; 2 Laboratory of Cancer Genetics (Lageca), School of Medical Sciences, University of Campinas (Unicamp), Campinas 13083-887, SP, Brazil; 3 Gastroenterology Unit, School of Medical Sciences, University of Campinas (Unicamp), Campinas 13083-887, SP, Brazil; 4 Simulation Laboratory, School of Applied Sciences, University of Campinas, Limeira 13484-350, SP, Brazil

**Keywords:** Crohn’s disease, adalimumab, immunogenicity, immune complexes, artificial intelligence

## Abstract

**Background:** The development of anti-drug antibodies (ADAs) and resulting immune complexes are key mechanisms behind the secondary loss of response to adalimumab in Crohn’s disease (CD). Despite their clinical importance, routine immunogenicity assays are limited, underscoring the need for alternative predictive approaches. **Objective:** This study aimed to develop interpretable artificial neural network (ANN) models to predict immune complex formation and estimate serum adalimumab levels using routinely available clinical and laboratory data from CD patients. **Methods:** A prospective analysis was performed on 58 CD patients on maintenance adalimumab. Immune complexes and serum adalimumab were measured via ELISA and lateral flow assays. ANN and ensemble regression models were trained on demographic, clinical, and inflammatory data, with performance evaluated by five-fold cross-validation. Interpretability was enhanced using Garson’s algorithm and permutation importance. **Results:** The ANN-based classification model accurately predicted ADA immune complex formation, achieving an accuracy of 77.47% and an area under the curve (AUC) of 82.63%. The main predictive variables included extraintestinal manifestations, perianal disease, disease behavior, and age at diagnosis. For estimating serum adalimumab levels measured by ELISA, the model performed modestly (accuracy 59.89%, AUC 79.72%), incorporating factors such as Montreal classification, perianal disease, C-reactive protein, immunosuppressant use, and disease duration. **Conclusions:** Interpretable ANN models robustly predict anti-adalimumab immune complexes and, to a lesser extent, serum adalimumab, using clinically available data, including perianal disease. This proof-of-concept study is limited by the relatively small, single-center dataset (n = 58), which may affect model generalizability and increase the risk of overfitting. External validation in larger and multicenter cohorts is required before clinical implementation.

## 1. Introduction

Inflammatory bowel diseases (IBDs), which primarily include Crohn’s disease (CD) and ulcerative colitis (UC), are chronic, relapsing disorders affecting the gastrointestinal tract [1]. These conditions are characterized by transmural intestinal inflammation in CD and superficial inflammation in UC. Their pathogenesis is multifactorial, involving genetic susceptibility, alterations in the intestinal microbiota, impairment of the mucosal barrier, and dysregulated immune responses [1]. Clinically, CD manifests with periods of disease activity and remission, potentially progressing to complications such as strictures, fistulas, abscesses, hemorrhage, malnutrition, and, in severe cases, necessitating surgical intervention [2].

In recent decades, therapeutic strategies for CD have undergone significant advancements with the introduction of biologics, particularly monoclonal antibodies targeting tumor necrosis factor-alpha (TNF-α), such as infliximab and adalimumab [3]. These agents have improved disease management by enhancing inflammatory control, supporting mucosal healing, and reducing hospitalizations and surgical interventions [3,4]. Adalimumab stands out among biologics for its subcutaneous administration, predictable pharmacokinetics, and favorable safety profile, which support its widespread use as a maintenance therapy [5].

Despite the notable clinical benefits associated with adalimumab use, a considerable number of patients eventually exhibit a loss of therapeutic response (LOR). This can be attributed to a range of pharmacokinetic and immunological mechanisms, among which the development of anti-drug antibodies (ADAs) plays a central role [6,7]. The emergence of ADAs may result in the formation of immune complexes, which can enhance drug clearance, lower serum drug levels, and ultimately diminish clinical efficacy [8]. Immune complexes are linked to LOR and may trigger type III hypersensitivity through tissue deposition, complement activation, and neutrophil recruitment. Larger complexes can also cause infusion reactions via cytokine release [9,10,11]. Timely identification of this immunogenic response is essential to prevent therapeutic failure and to inform strategies for treatment adjustment and optimization.

In contemporary clinical practice, the management of ADA development generally relies on therapeutic drug monitoring (TDM), dose optimization, switching within or outside the anti-TNF class, and the use of concomitant immunosuppressants to mitigate immunogenicity. These treatment pathways underscore the ongoing need for predictive tools that can anticipate ADA formation before loss of response occurs [12,13].

Nonetheless, the evaluation of immunogenicity continues to depend on specialized laboratory techniques, including ELISA assays, which are often unavailable in routine clinical settings and demand specific processing time and infrastructure. Therefore, there is an increasing demand for developing predictive methods that can estimate immune complex formation and serum drug levels using readily accessible and commonly collected clinical and laboratory parameters.

In this context, the use of artificial intelligence (AI) approaches, particularly artificial neural networks (ANNs), has garnered increasing attention within the field of personalized medicine. These methodologies are capable of detecting intricate patterns and non-linear relationships among numerous clinical variables, frequently overcoming the constraints inherent to conventional statistical techniques [14,15]. Although the use of ANN has expanded considerably across multiple medical domains, including IBD to determine disease prognosis and predict treatment response [16], their integration into IBD research and clinical decision-making related to drug monitoring remains relatively underexplored.

Beyond gastroenterology, AI, particularly ANN-based methodologies, has already demonstrated meaningful clinical impact across several medical specialties. In radiology and pathology, deep learning models have been applied to assist image interpretation, reduce interobserver variability, and improve workflow efficiency [17,18]. Similarly, in dermatology and ophthalmology, ANNs are now routinely integrated into automated lesion-detection and screening programs, demonstrating their safe and effective incorporation into clinical practice [19,20]. Although the adoption of AI in IBD remains at an earlier stage, the successful implementation of neural network systems in other fields highlights their potential to enhance diagnostic precision, minimize delays in clinical assessment, and support individualized therapeutic decision-making [21]. As these technologies become increasingly embedded in healthcare systems, their application in IBD is expected to follow a similar trajectory toward broader clinical utility.

Emerging studies have highlighted the utility of ANNs in supporting tasks such as disease phenotyping, predicting endoscopic activity, and therapeutic response models through the combination of clinical, biochemical, and imaging parameters [14,22,23,24]. While some machine learning approaches have addressed the prediction of loss of response to anti-TNF agents, including infliximab [14], to our knowledge, no prior studies have specifically applied neural network models to predict the development of immune complexes and serum biological levels in CD patients receiving adalimumab.

ANN was selected over other machine learning approaches because they are particularly suited to capturing nonlinear and multidimensional relationships among clinical variables, as expected in complex immunogenicity pathways. Moreover, prior studies in IBD have demonstrated the robust performance of ANN-based models in diverse predictive applications, further supporting their use in this context.

Given the clinical importance of immunogenicity and the availability of structured clinical data in routine care, ANNs represent a compelling approach for early risk stratification. Additionally, interpretable models may provide insight into which patient-specific variables most strongly contribute to immune activation, ultimately supporting more personalized and proactive treatment strategies in the management of CD.

Thus, this study aimed to develop and evaluate ANN models capable of accurately predicting the formation of immune complexes in patients with CD treated with adalimumab. In addition, we aimed to identify and interpret the most influential clinical and laboratory variables that contribute to these predictions, thereby enhancing personalized therapeutic decision-making in clinical practice.

## 2. Materials and Methods

### 2.1. Cohort

This was a prospective cross-sectional study. The present analysis included a cross-sectional observational study conducted between 2020 and 2023 that enrolled 58 individuals diagnosed with CD. Eligibility criteria included patients with a confirmed diagnosis of CD, based on clinical and endoscopic findings, who were receiving ongoing maintenance therapy with adalimumab for at least two months. Consistent attendance at routine follow-up appointments was also required. Based on predefined objective criteria, patients were stratified into either the active disease or remission groups. Disease activity was determined through colonoscopy, where active disease was defined as a Crohn’s Disease Endoscopic Index of Severity (CDEIS) score of 5 or higher, or the presence of deep ulcers in at least one intestinal segment, or by magnetic resonance enterography (MRE), in which active disease was indicated by the presence of deep ulcers in at least one intestinal segment.

All enrolled participants provided written informed consent before their inclusion. The study protocol was approved by the local Research Ethics Committee (CAAE no. 92894418.4.0000.5404) and conducted following the principles outlined in the Strengthening the Reporting of Observational Studies in Epidemiology (STROBE) guidelines [25].

### 2.2. Artificial Neural Network Modeling

Three types of predictive models were developed using artificial neural networks: classification models to predict the formation of immune complexes measured by ELISA assay (Bühlmann^®^ CIC-CIQ EIA—Bühlmann Laboratories AG, Schönenbuch, Switzerland), and regression models to estimate serum adalimumab concentrations measured by lateral flow assay (Bühlmann^®^ Quantum rapid test—Blue—Bühlmann Laboratories AG, Schönenbuch, Switzerland), and by ELISA assay (Promonitor^®^—Progenika Biopharma, S.A., Derio, Spain). The measurements of adalimumab and immunocomplex serum levels were according to the manufacturer’s instructions.

### 2.3. Software and Computational Environment

The computational framework was developed in Python (version 3.12.12 (main, 10 October 2025, 08:52:57) [GCC 11.4.0]), leveraging key open-source libraries, including Scikit-learn (1.6.1) for model implementation, Pandas (version 2.2.2) and NumPy (version 2.0.2) for data manipulation, and Matplotlib (version 3.10.0) and Seaborn (version 0.13.2) for visualization. Hyperparameter optimization was performed using Optuna (version 4.6.0) [26], an open-source framework that employs a define-by-run API to dynamically construct the search space. A total of 30 trials were conducted to maximize the Macro F1-Score, tuning critical parameters such as the number of features to select (“k_best”, range: 5 to the minimum of 20 or the total number of numerical features), the maximum number of features used by the model (“max_features”, range: 3 to the number of selected numerical features), and “model selection” (from predefined algorithms including LogisticRegression, RandomForest, MLPClassifier, and ensemble methods). Optimization was carried out using 3-fold stratified cross-validation to ensure robust parameter selection.

### 2.4. Data Preprocessing

Clinical, laboratory, and demographic data from patients with CD were subjected to a standardized preprocessing pipeline to ensure data integrity, comparability across variables, and optimal model performance. This automated routine was implemented through modularized functions, encompassing data imputation, categorical encoding, and normalization steps. For continuous variables (such as age at diagnosis, CDAI, and duration of adalimumab therapy), missing values were imputed using the median. This robust approach mitigates the influence of outliers. Subsequently, all continuous features were normalized using z-score scaling, which facilitates stable convergence during model training by aligning the scales of heterogeneous features. Categorical variables (including sex, presence of extraintestinal manifestations, perianal disease, and Montreal classification) were transformed into binary vectors. This step ensured compatibility with ANN architectures, which require numerical inputs, while preserving the categorical nature of the information without introducing artificial ordinality. No substantial class imbalance was present (immune complex: 38 positives vs. 32 negatives). All normalization and imputation steps were performed within each training fold to prevent data leakage during cross-validation.

### 2.5. Feature Selection

Feature selection was performed using the SelectKBest method, based on analysis of variance (ANOVA) via the f_classif function, to identify the most informative numerical variables associated with the outcome of interest. This process was controlled via the Optuna optimization library, which automated the search for the optimal combination of explanatory variables, thereby enhancing model efficiency and reducing manual selection bias. For the classification model, univariate analysis was employed to identify the most relevant predictors of immune complex formation. Four features were retained: presence of extraintestinal manifestations, CDAI-based disease activity, perianal disease involvement, and disease behavior according to the Montreal classification. In contrast, the regression models incorporated broader features to capture the multifactorial nature of inflammation and therapeutic response in CD.

### 2.6. Model Development and Validation

Before model training, the dataset was partitioned into training and testing sets in a 70:30 ratio, maintaining class stratification to preserve the distribution of the target variable. Stratified k-fold cross-validation was then employed during hyperparameter optimization and performance evaluation, ensuring that the predictive models remained robust and generalizable to unseen data. A 70:30 train-test split was applied prior to cross-validation. All preprocessing steps, including normalization, encoding, and imputation, were conducted within each fold to prevent data leakage. Five-fold stratified cross-validation was performed exclusively on the training set during hyperparameter optimization.

The classification model was based on a multilayer perceptron neural network with one hidden layer and a hyperbolic tangent activation function. The ANN architecture was implemented as a multilayer perceptron comprising one to three hidden layers, with the optimal configuration determined via Bayesian hyperparameter search. Each hidden layer contained 16–64 neurons, and activation functions assessed included hyperbolic tangent and ReLU. Models were trained for up to 300 epochs, with early stopping employed based on validation loss to prevent overfitting.

Training used an adaptive gradient descent optimizer with early stopping criteria to prevent overfitting. Regression models included both multilayer neural networks and ensemble-based methods (Random Forest and XGBoost), with hyperparameter tuning performed via Bayesian optimization. Model performance was evaluated using five-fold cross-validation. For classification, performance metrics included accuracy, macro-averaged precision, recall, F1 Score, and area under the receiver operating characteristic curve (AUC). For regression models, the coefficient of determination (R^2^) and mean absolute error (MAE) were used to assess predictive performance.

Given the modest dataset size, particular attention was given to mitigating overfitting through early stopping, dropout regularization, Bayesian hyperparameter optimization, and nested cross-validation.

### 2.7. Model Interpretability

To enhance clinical interpretability, feature importance analyses were conducted. For the classification model, Garson’s algorithm was applied to assess the relative contribution of each input variable. In tree-based regression models, intrinsic feature importance scores were extracted. For neural network regressors, permutation-based importance metrics and partial dependence plots were employed to investigate the impact of individual predictors on model outputs.

To address the interpretability limitations of neural network models, which are particularly important in clinical applications [27], we used Garson’s algorithm [28] for the MLP classifier. This approach evaluates the network’s connection weights to assess the relative contribution of each predictor, providing valuable insights into the model’s decision-making process and improving its clinical transparency.

### 2.8. Data Availability Statement

The complete code is provided in the Supplementary Materials. The code covers the entire analytical workflow, including data preprocessing, feature selection, hyperparameter optimization using Optuna (version 4.6.0), model training and evaluation, and interpretability analysis using Garson’s algorithm.

## 3. Results

The predictive tasks were designed to support clinical decision-making in the context of CD, particularly regarding immune response, inflammatory burden, and treatment efficacy. Three primary outcomes were modeled: (1) immune complex formation, (2) adalimumab’s serum level measured by lateral flow assay, and (3) adalimumab’s serum level measured by ELISA assay. Hyperparameter optimization was conducted using Optuna.

### 3.1. Clinical and Demographic Data

A total of 58 patients with CD were included in this study. The clinical and demographic characteristics of the cohort are presented in Table 1. The median age was 42 years, with 26 male and 32 female patients. The median disease duration was 12 months.

According to the Montreal classification, the age at diagnosis was A1 in 7 patients, A2 in 38 patients, and A3 in 13 patients. Disease location was classified as L1 in 15 patients, L2 in 17, and L3 in 26. Disease behavior was B1 in 30 patients, B2 in 14, and B3 in 14.

Perianal disease was present in 20 patients, and extraintestinal manifestations were reported in 32 patients. Two patients were active smokers. Concomitant immunosuppressant therapy was used by 27 patients.

Active disease was observed in 36 patients, while 22 patients were in remission. Previous CD-related surgeries were documented in 34 patients. The median duration of adalimumab therapy was 60 months. A total of 16 patients had previously been treated with another anti-TNFα agent. Adverse reactions to adalimumab were reported in 4 patients.

### 3.2. Prediction of Anti-Adalimumab Immune Complex Formation

**Figure 1:**
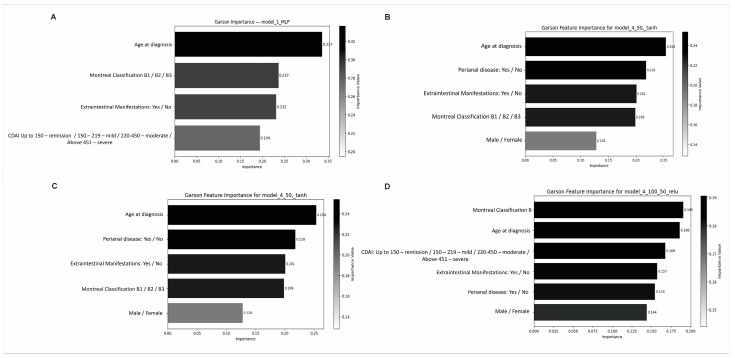
Neural network-based predictive models were developed using an integrated set of clinical, laboratory, and epidemiological variables, with immune complex formation defined as the outcome variable. Dimensionless percentage contribution of each variable to the predictive model, based on the combined analysis of the following variables: (**A**) Age at Diagnosis, Montreal Classification B1/B2/B3, Extraintestinal Manifestations, and CDAI. (**B**) Age at Diagnosis, Montreal Classification B1/B2/B3, Perineal Disease, Extraintestinal Manifestations, and Sex. (**C**) Age at diagnosis, Perianal disease, Extra-intestinal Manifestations, Montreal Classification B1/B2/B3, Sex. (**D**) Montreal Classification B1/B2/B3, Age at diagnosis, CDAI, Extraintestinal Manifestations, Perianal disease, and Sex. CDAI: Crohn’s Disease Activity Index.

The prediction of anti-adalimumab immune complex formation was addressed using multiple supervised learning models trained on diverse sets of clinically meaningful variables (Table 2 and Figure 1). The most accurate model incorporated age at diagnosis, Montreal’s Classification “Behaviour”, extraintestinal manifestations, and CDAI. This configuration achieved the highest overall accuracy (77.4%) and a robust area under the receiver operating characteristic curve (AUC-ROC-OVR: 82.63%), indicating a strong discriminative capacity (Table 2; Figure 1A).

A model integrating Montreal classification, age-related variables, and inflammatory features, including the presence of extraintestinal and perianal disease, also yielded strong classification performance (Figure 1D), with an accuracy of 77.29%. Importantly, this model demonstrated a superior ROC-AUC-OVR (82.73%) (Table 2).

### 3.3. Prediction of Adalimumab’s Serum Level Measured by Lateral Flow Assay

Despite testing various machine learning algorithms and data preprocessing strategies, none of the constructed models achieved an acceptable level of accuracy or generalizability in predicting adalimumab serum levels as measured by the lateral flow assay. Performance metrics indicated poor fit and low predictive power across all approaches. The clinical variables included in the models did not provide a sufficient signal to predict serum concentrations. Additionally, model performance did not improve with feature selection, scaling, or hyperparameter tuning. These findings suggest that the available clinical and laboratory variables alone cannot accurately estimate adalimumab serum levels using lateral flow assays.

### 3.4. Prediction of Adalimumab’s Serum Level Measured by ELISA

**Figure 2:**
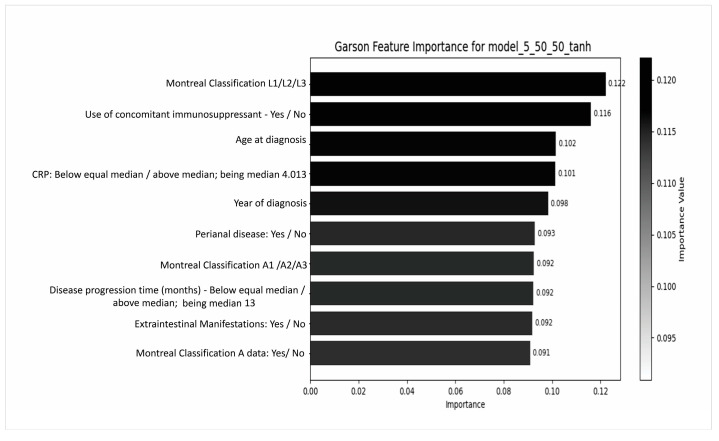
Predictive models based on neural networks were constructed using a comprehensive set of clinical, laboratory, and epidemiological variables, with serum adalimumab concentrations measured by ELISA assay defined as the outcome variable. Dimensionless percentage contribution of each variable to the predictive model, based on the combined analysis of the following variables: Montreal Classification L1/L2/L3, Use of concomitant immunosuppressant, Age of diagnosis, CRP, Year of diagnosis, Perineal Disease, Montreal Classification A1/A2/A3, Disease progression in months, Extra-intestinal Manifestations, Montreal Classification A. CRP: C-reactive protein.

The third objective aimed to estimate adalimumab’s serum level compared to that measured by the ELISA assay (Promonitor^®^) (Table 3 and Figure 2). Serum adalimumab concentrations (μg/mL) were categorized into two classes based on the assay manufacturer’s reference range: therapeutic (≥5.0 μg/mL) and subtherapeutic (<5.0 μg/mL). These categories were used as the target classes for the classification model. We report the model’s performance in relation to this binarized outcome.

The only performing model integrated Montreal Classification (A1/A2/A3), CRP levels, use of concomitant immunosuppressants, year of diagnosis, and Montreal classifications L and A, along with perianal and extraintestinal manifestations and age at diagnosis. This configuration achieved an overall accuracy of 59.89% and a notably high F1-score (79.72%), reflecting a balanced classification performance. Despite its moderate accuracy, the model displayed a robust ROC-AUC-OVR of 82.63%, indicating a strong discriminative ability to predict adalimumab serum levels.

Although the models demonstrated promising discriminative performance, these findings should be regarded as preliminary. The dataset remains relatively modest for deep learning applications, and external validation will be essential before clinical translation is feasible.

## 4. Discussion

In this study, we developed and evaluated AI-based models, specifically ANN, to predict immunogenicity and drug serum levels in patients with CD undergoing maintenance therapy with adalimumab. The models aimed to identify individuals at risk of forming anti-adalimumab immune complexes and to estimate serum drug concentrations as determined by lateral flow and ELISA assays.

The expanding incorporation of ANN–based tools into routine clinical workflows across multiple medical specialties provides a relevant framework for interpreting our findings. In radiology [17], for instance, AI systems have been integrated into standard mammography and chest imaging workflows, enhancing detection rates while alleviating clinician workload. In digital pathology [18], ANNs contribute to tumor grading and the identification of molecular signatures from histologic slides, thereby supporting pathologists in complex diagnostic evaluations. Comparable advances have been observed in dermatology and ophthalmology [19,20], where neural models are used for triage, risk stratification, and real-time decision support. Although IBD care has not yet reached a comparable level of technological adoption, successful implementations in other fields demonstrate that neural networks can be deployed safely, validated prospectively, and embedded in clinical decision-support systems. As healthcare increasingly advances toward precision medicine, the diffusion of these approaches underscores their potential to enhance therapeutic monitoring, optimize resource allocation, and support individualized treatment strategies for patients with IBD.

In our study, the classification model with the highest performance for predicting immunocomplexes of adalimumab achieved an overall accuracy of 77.47% and an AUC of 82.63%, leveraging routinely collected clinical variables such as smoking status, age at collection, sex, perianal disease involvement, and the presence of extraintestinal manifestations. These findings underscore the feasibility of predicting immunocomplex formation using accessible clinical data, offering a potential solution in settings where advanced immunogenicity assays are unavailable.

Other models also demonstrated strong predictive capabilities (accuracies ranging from 77.29%) for immune complexes, particularly those incorporating Montreal classification and CDAI variables. Importantly, perianal disease and extraintestinal manifestations were consistently ranked among the top predictors, reinforcing their association with systemic immune dysregulation and aggressive disease phenotypes. These observations support the biological plausibility of the models and align with the existing literature, which describes their role in heightened immune activation and tissue barrier dysfunction [29,30,31,32].

These results are especially encouraging considering that previously published studies have primarily applied AI to endoscopic examinations and image analysis, rather than to this specific predictive modeling approach in IBD [33,34,35,36,37]. Our study contributes a more targeted and clinically meaningful endpoint: the formation of anti-adalimumab immune complexes, a recognized mechanism underlying secondary loss of response and diminished drug efficacy [8].

Regarding the prediction of serum adalimumab concentrations, regression models yielded variable performance. For the ELISA assay, the top-performing model achieved 59.89% accuracy and an AUC of 79.72%, indicating good discriminative power. This model incorporated a comprehensive set of clinical and inflammatory factors, including Montreal’s Classification age, CRP, use of concomitant immunosuppressant, year of diagnosis, Montreal’s Classification (“Localization”), perineal disease, disease duration, and extra-intestinal manifestations. This is clinically relevant, as serum adalimumab levels correlate strongly with treatment efficacy, mucosal healing, and the prevention of relapse [38,39,40,41].

In contrast, none of the models developed to predict concentrations obtained via lateral flow assay yielded satisfactory results. Despite testing multiple machine learning algorithms and implementing various preprocessing strategies, the models consistently failed to achieve acceptable accuracy or generalizability. These findings suggest that the available clinical variables did not provide a sufficient predictive signal to accurately estimate serum concentrations measured by rapid testing (lateral flow assay). The limited performance of models predicting lateral flow assay results likely reflects the intrinsic analytical variability and narrower dynamic range of rapid tests compared with ELISA. These technical constraints reduce the detectable signal from clinical variables and challenge the development of accurate predictive models, thereby limiting machine-learning models’ ability to identify meaningful patterns.

This outcome carries significant implications for clinical practice. The inability to develop reliable predictive models for lateral flow assay results highlights the continued need to use these rapid diagnostic tools directly in TDM. Although AI offers promising avenues for anticipating drug exposure and guiding treatment decisions, our results highlight that, at present, rapid assays remain indispensable for real-time assessment of adalimumab levels. Consequently, their integration into routine care remains essential, particularly in settings where timely therapeutic adjustments are critical and access to ELISA-based quantification is limited.

Predictive modeling of immunogenicity and drug exposure through non-invasive, routinely collected variables can support TDM without requiring costly or resource-intensive laboratory assays. Such models may facilitate the earlier identification of patients at risk for subtherapeutic drug levels or treatment failure, thereby informing timely interventions, such as dose adjustments or therapy switches [42,43,44].

A significant strength of our approach lies in the biological and clinical plausibility of the predictors identified through the model. Rather than relying solely on abstract features, the variables we utilize align with established clinical understanding of CD’s pathophysiology and patient phenotyping. Specifically, the presence of extraintestinal manifestations reflects systemic immune dysregulation [45,46,47]. Perianal disease involvement [48,49], often associated with more aggressive or refractory disease courses, may similarly reflect heightened immune activation or barrier dysfunction that contributes to immunogenicity. Moreover, disease behavior as classified by the Montreal system (inflammatory, stricturing, or penetrating) provides insight into the chronicity and tissue-destructive potential of the disease, potentially influencing antigen exposure and immune priming [50,51,52].

The integration of AI into IBD management exemplifies a broader trend toward precision medicine, where treatment plans are tailored to individual patient characteristics and needs. Traditional statistical models often face limitations in capturing the complex, multidimensional, and nonlinear interdependencies among clinical variables, especially in relatively small and heterogeneous cohorts. In contrast, ANNs provide a flexible framework that can model these intricate associations and higher-order interactions, reflecting the complexity of biological systems.

Importantly, our study prioritized the interpretability of the AI models. By employing techniques such as Garson’s algorithm, permutation importance metrics, and partial dependence plots, we were able to dissect the ANN outputs and quantify the relative importance of each predictor. This tool was selected to elucidate the “black box” nature of the model and provide insights into predictor importance, thereby fulfilling the objectives of transparency and interpretability. Providing interpretable predictions fosters trust and facilitates the translation of AI tools into routine clinical practice.

To our knowledge, this is the first study to apply interpretable ANN models to the prediction of anti-adalimumab immune complexes in CD. The integration of multidimensional real-world data (clinical, biochemical, and genetic) enhances the generalizability and relevance of the findings. Moreover, the methodological rigor, encompassing systematic preprocessing, robust feature selection, Bayesian hyperparameter tuning, and stratified five-fold cross-validation, supports the reliability and reproducibility of the models.

From a health-technology assessment perspective, ANN-based predictive tools have the potential to reduce reliance on costly immunogenicity assays, shorten decision-making timelines within therapeutic drug monitoring, and improve resource allocation by enabling earlier identification of patients at risk for treatment failure. Such approaches may contribute to greater sustainability in healthcare delivery, particularly in settings with limited laboratory infrastructure.

Quantification of adalimumab via ELISA and detection of immune complexes were conducted directly on serum samples of the patients included in the study. This direct integration of laboratory assays with clinical data ensured an important internal consistency, thereby enhancing the translational relevance of our predictive models. Notably, the models’ performance in predicting immune complex formation and estimating serum drug concentrations, as measured by ELISA, demonstrated strong concordance with the empirical bench data.

**Figure 3:**
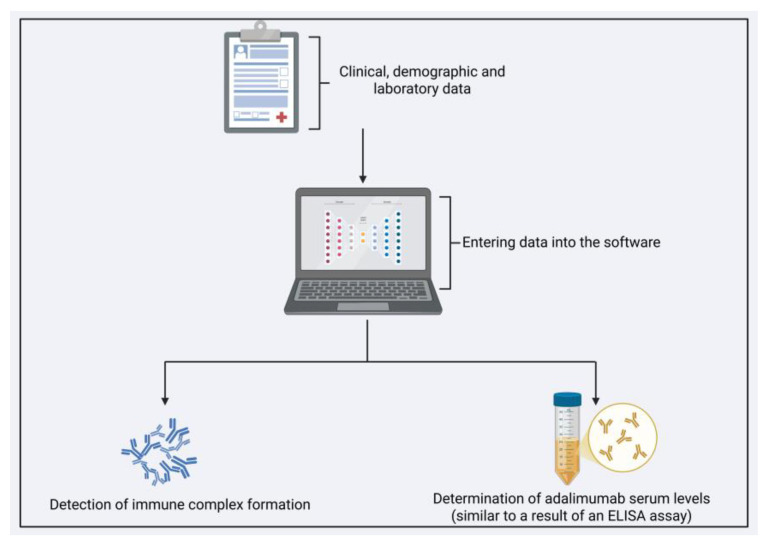
Workflow for Predictive Modeling of Adalimumab Monitoring in Clinical Practice. Integration of clinical, demographic, and laboratory data into a machine learning framework for the prediction of immune complex formation and serum adalimumab levels in Crohn’s disease patients undergoing adalimumab therapy. Created in BioRender. Velloso, L. (2025) https://BioRender.com/kcuwxbc (accessed on 25 November 2025). Taken together, our findings highlight the potential of neural network–based approaches to support therapeutic monitoring and individualized treatment decisions in CD. However, this study has limitations that must be acknowledged. The modest sample size may reduce model stability and limit the detection of more subtle nonlinear interactions among clinical and laboratory variables. Additionally, the single-center design restricts population heterogeneity and may limit the generalizability of the models to different clinical settings. Although the proposed models demonstrated promising performance, external validation in larger, multicenter cohorts is essential to verify their robustness and clinical applicability. Assay-related variability—particularly the differences in dynamic range and analytical precision between ELISA and lateral flow methods—may also have influenced the predictive performance, especially for rapid-test–based models. These considerations emphasize the preliminary, proof-of-concept nature of our study and underscore the need for broader validation before these models can be incorporated into routine clinical practice.

By employing both classification and regression architectures, we addressed complementary aspects of immunogenicity: the binary risk assessment of immune complex development and continuous estimation of drug levels (Figure 3). These outputs provide distinct yet synergistic clinical benefits, supporting risk stratification, therapeutic optimization, and proactive clinical management.

Future studies should incorporate multicenter datasets encompassing greater heterogeneity in disease phenotypes and treatment exposures to enhance model robustness and improve clinical applicability.

## 5. Conclusions

In conclusion, our study demonstrates the feasibility and clinical potential of using interpretable ANN models to predict anti-adalimumab immune complex formation and serum drug levels, which are similar to those assessed by ELISA, in CD patients undergoing adalimumab. These findings represent a step forward in applying precision medicine principles to IBD management and highlight the transformative role of AI in tailoring biologic therapy. With continued refinement and validation, such tools may contribute to more personalized, cost-effective, and outcome-driven care for patients with IBD.

## Figures and Tables

**Table 1:** Demographic and clinical characteristics of the participants of the study.

	CD Patients
Number of patients	58
Gender (M/F)	26/32
Age (years)	42 (8–79)
Disease duration (months)	12 (1–37)
Active disease (yes/no)	36/22
Previous surgeries (yes/no)	34/36
Immunosuppressant use (yes/no)	27/31
Age at diagnosis (A1/A2/A3)	7/38/13
Location (L1/L2/L3)	15/17/26
Behavior (B1/B2/B3)	30/14/14
Perianal disease (yes/no)	20/36
Extraintestinal manifestations (yes/no)	32/26
Smoking (yes/no)	2/56
Duration of adalimumab therapy (months)	60 (2–216)
Presentation of adverse reaction to adalimumab (yes/no)	4/54
Previous anti-TNFα therapy (yes/no)	16/42

Numerical variables are described by their median (minimum, maximum), and categorical variables by their absolute frequencies. M = male, F = female.

**Table 2:** Model Performance for Predicting Anti-Adalimumab Immune Complexes.

Selected_Features	Accuracy	Precision Macro	Recall Macro	F1 Macro	ROC_AUC_OVR
[‘Age at Diagnosis’. ‘Montreal Classification B1/B2/B3’. ‘Extraintestinal Manifestations: Yes/No’. ‘CDAI: Up to 150—remission/150–219—mild/220—450 moderate/Above 451—severe’]	77.47%	61.15%	69.75%	63.14%	82.63%
[‘Age at Diagnosis’. ‘Perineal Disease: Yes/No’. ‘Male/Female’. ‘Extraintestinal Manifestations: Yes/No’, ‘Montreal Classification B1/B2/B3’, ‘Sex’]	77.29%	60.73%	61.82%	60.85%	81.82%
[‘Age at diagnosis’. ‘Perineal Disease: Yes/No’. ‘Extraintestinal manifestations: Yes/No’. ‘Montreal Classification B1/B2/B3’. ‘Sex’]	77.29%	60.73%	61.82%	60.85%	80.20%
[Montreal Classification B’. ‘Age at diagnosis’. ‘CDAI: ‘Up to 150—remission/150–219—mild/220–450 moderate/Above 451—severe’. ‘Extraintestinal Manifestations: Yes/No’. ‘Perianal disease: Yes/No’, ‘Male/Female’]	77.29%	57.97%	61.82%	59.64%	82.73%

**Table 3:** Model Performance for Predicting Adalimumab Serum Levels by ELISA Assay.

Selected_Features	Accuracy	Precision Macro	Recall Macro	F1 Macro	ROC_AUC_OVR
‘Montreal Classification L1/L2/L3’. ‘Use of concomitant immunosuppressant: Yes/No’; ‘CRP: Below or equal to median/Above median—with median being 4.035’. ‘Year of diagnosis’. ‘Perineal Disease: Yes/No’. ‘Montreal Classification A1/A2/A3’. ‘Disease progression: Below-equal to median/Above median—with median being 13’. ‘Extra-intestinal Manifestations: Yes/No’. ‘Montreal Classification A’.	59.89%	58.15%	56.61%	53.05	79.72%

## Data Availability

The original contributions presented in this study are included in the article. Further inquiries can be directed to the corresponding author.

## Supplementary Materials

The following supporting information can be downloaded at: https://www.mdpi.com/article/10.3390/pharmaceutics17121536/s1, File S1: Computational Scripts.

## Abbreviations

The following abbreviations are used in this manuscript:

A1/A2/A3	Age at Diagnosis Categories (Montreal Classification)
ADA	Anti-Drug Antibody
AI	Artificial Intelligence
ANN	Artificial Neural Network
AUC	Area Under the Curve
B	Behavior (Montreal Classification)
B1/B2/B3	Behavior Categories (Montreal Classification)
CDAI	Crohn’s Disease Activity Index
CD	Crohn’s Disease
CDEIS	Crohn’s Disease Endoscopic Index of Severity
CRP	C-Reactive Protein
DII	Doença Inflamatória Intestinal (Inflammatory Bowel Disease)
ELISA	Enzyme-Linked Immunosorbent Assay
IBD	Inflammatory Bowel Disease
IC	Immune Complex
L	Location (Montreal Classification)
L1/L2/L3	Location Categories (Montreal Classification)
LOR	Loss of Response
MAE	Mean Absolute Error
MLP	Multilayer Perceptron
MRE	Magnetic Resonance Enterography
Optuna	Optimization Framework for Hyperparameter Tuning
R^2^	Coefficient of Determination
ROC	Receiver Operating Characteristic
STROBE	Strengthening the Reporting of Observational Studies in Epidemiology
TDM	Therapeutic Drug Monitoring
TNF-α	Tumor Necrosis Factor-alpha
UC	Ulcerative Colitis
