# Supplementary material for: Interpretable Artificial Neural Network Models for Predicting Anti-Adalimumab Immune Complex and Serum Drug Level in Crohn’s Disease: A Proof-of-Concept Study
Source: Pharmaceutics. 2025 Nov 29;17(12):1536. doi: 10.3390/pharmaceutics17121536 (PMC12735940; doi:10.3390/pharmaceutics17121536)
Supplement: Supplementary file 1 [file pharmaceutics-17-01536-s001.zip › pharmaceutics-3971051-supplementary.pdf]

Supplementary Material: Computational Scripts

```
# === Instalações (se necessário no Colab) ===
!pip install -q optuna

# === IMPORTS ===
import os
import random
import pandas as pd
import numpy as np
import optuna
import matplotlib.pyplot as plt
import seaborn as sns

from tqdm.notebook import tqdm
from sklearn.model_selection import train_test_split, StratifiedKFold,
cross_validate
from sklearn.preprocessing import StandardScaler, OneHotEncoder
from sklearn.impute import SimpleImputer
from sklearn.pipeline import Pipeline
from sklearn.compose import ColumnTransformer
from sklearn.feature_selection import SelectKBest, f_classif
from sklearn.metrics import classification_report, confusion_matrix,
roc_auc_score, roc_curve

from sklearn.linear_model import LogisticRegression
from sklearn.svm import SVC
from sklearn.neighbors import KNeighborsClassifier
from sklearn.tree import DecisionTreeClassifier
from sklearn.ensemble import (
    RandomForestClassifier, GradientBoostingClassifier,
    VotingClassifier, StackingClassifier
)
from sklearn.naive_bayes import GaussianNB
from sklearn.neural_network import MLPClassifier

# === DEFINIÇÃO DOS MODELOS ===
models = {
    'LogisticRegression': LogisticRegression(max_iter=1000),
    'SVC': SVC(probability=True),
    'KNN': KNeighborsClassifier(),
    'DecisionTree': DecisionTreeClassifier(),
    'RandomForest': RandomForestClassifier(),
```

```python
    'GradientBoosting': GradientBoostingClassifier(),
    'GaussianNB': GaussianNB(),
    'MLP': MLPClassifier(max_iter=1000)
}

# === ENSEMBLES ===
models['Voting'] = VotingClassifier(estimators=[
    ('lr', LogisticRegression(max_iter=1000)),
    ('rf', RandomForestClassifier()),
    ('svc', SVC(probability=True))
], voting='soft')

models['Stacking'] = StackingClassifier(estimators=[
    ('rf', RandomForestClassifier()),
    ('gb', GradientBoostingClassifier()),
    ('svc', SVC(probability=True))
], final_estimator=LogisticRegression())

# === MÉTRICAS DE AVALIAÇÃO ===
scoring = ['accuracy', 'precision_macro', 'recall_macro', 'f1_macro',
'roc_auc_ovr']

# === PRÉ-PROCESSADOR ===
def make_preprocessor(num_cols, cat_cols):
    transformers = []
    if num_cols:
        transformers.append(('num', Pipeline([
            ('imputer', SimpleImputer(strategy='median')),
            ('scaler', StandardScaler())
        ]), num_cols))
    if cat_cols:
        transformers.append(('cat',
OneHotEncoder(handle_unknown='ignore'), cat_cols))
    return ColumnTransformer(transformers)

# === GARSON PARA MLP ===
def compute_garson_importance(pipeline, numerical_features, model_name,
fig_dir):
    print(f"Executando Garson para: {model_name}")
    try:
        mlp_model = pipeline.named_steps['model']
        coefs = mlp_model.coefs_
```

```
        if len(coefs) < 2:
            return pd.DataFrame()

        input_hidden_weights = np.abs(coefs[0])
        hidden_output_weights = np.abs(coefs[1])

        if hidden_output_weights.ndim == 2:
            hidden_output_weights = hidden_output_weights.sum(axis=1)

        garson_weights = (input_hidden_weights @
hidden_output_weights).ravel()
        garson_weights /= garson_weights.sum()

        if len(numerical_features) != len(garson_weights):
            return pd.DataFrame()

        df = pd.DataFrame({
            'Feature': numerical_features,
            'GarsonImportance': garson_weights
        }).sort_values('GarsonImportance', ascending=False)

        fig, ax = plt.subplots(figsize=(10, 6))
        cmap = plt.cm.binary
        colors = cmap(df['GarsonImportance'] /
df['GarsonImportance'].max())
        bars = ax.barh(df['Feature'], df['GarsonImportance'],
color=colors)
        for bar, val in zip(bars, df['GarsonImportance']):
            ax.text(bar.get_width() + 0.001, bar.get_y() +
bar.get_height() / 2, f"{val:.3f}", va='center', ha='left')

        ax.invert_yaxis()
        ax.set_title(f'Garson Importance — {model_name}')
        ax.set_xlabel('Importance')
        sm = plt.cm.ScalarMappable(cmap=cmap,
norm=plt.Normalize(vmin=df['GarsonImportance'].min(),
vmax=df['GarsonImportance'].max()))
        sm.set_array([])
        plt.colorbar(sm, ax=ax).set_label('Importance Value')

        path = os.path.join(fig_dir,
f'{model_name}_garson_importance.png')
        fig.savefig(path, bbox_inches='tight')
```

```
        plt.close(fig)

        print(f"Salvo: {path}")
        return df

    except Exception as e:
        print(f"Erro no Garson: {e}")
        return pd.DataFrame()

# === DIVISÃO DE DADOS ===
X_train, X_test, y_train, y_test = train_test_split(X, y,
test_size=0.3, stratify=y, random_state=42)
num_cols = X.select_dtypes(include=np.number).columns.tolist()
cat_cols = X.select_dtypes(include='object').columns.tolist()

# === FUNÇÃO DO OPTUNA ===
configurations_testadas = []
def objective(trial):
    print("\nNovo trial:")
    k_best = trial.suggest_int('k_best', 5, min(20, len(num_cols)))
    selector = SelectKBest(f_classif, k=k_best).fit(X_train[num_cols],
y_train)
    selected_num_cols =
X_train[num_cols].columns[selector.get_support()].tolist()

    max_features = trial.suggest_int('max_features', 3,
len(selected_num_cols))
    selected_subset_num = random.sample(selected_num_cols,
max_features)
    selected_subset = selected_subset_num + cat_cols
    features_cat = [f for f in selected_subset if f in cat_cols]

    model_name = trial.suggest_categorical('model_name',
list(models.keys()))
    model = models[model_name]

    pipeline = Pipeline([
        ('preprocessor', make_preprocessor(selected_subset_num,
features_cat)),
        ('model', model)
    ])

    cv = StratifiedKFold(n_splits=3, shuffle=True, random_state=42)
```

```
    scores = cross_validate(pipeline, X_train[selected_subset],
y_train,
                            scoring=scoring, cv=cv,
return_train_score=False)

    avg_scores = {m: np.mean(scores[f'test_{m}']) for m in scoring}
    configurations_testadas.append({
        'params': trial.params,
        'selected_features': selected_subset,
        **avg_scores
    })

    return avg_scores['f1_macro']

# === RODAR OPTUNA ===
study = optuna.create_study(direction='maximize')
study.optimize(objective, n_trials=30)

df_results = pd.DataFrame(configurations_testadas)
df_top5 = df_results.sort_values(by='f1_macro',
ascending=False).head(5).reset_index(drop=True)

# === AVALIAR MODELOS FINAIS ===
def evaluate_top_models():
    final_report = []
    fig_dir = '/drive/My Drive/ArtigoLivia/figs'
    os.makedirs(fig_dir, exist_ok=True)

    for i, row in tqdm(df_top5.iterrows(), total=5):
        features = row['selected_features']
        features_num = [f for f in features if f in num_cols]
        features_cat = [f for f in features if f in cat_cols]

        model_name = row['params']['model_name']
        model = models[model_name]

        pipeline = Pipeline([
            ('preprocessor', make_preprocessor(features_num,
features_cat)),
            ('model', model)
        ])
        pipeline.fit(X_train[features], y_train)
        y_pred = pipeline.predict(X_test[features])
```

```
        y_proba = pipeline.predict_proba(X_test[features])[:, 1] if
hasattr(pipeline.named_steps['model'], 'predict_proba') and
len(np.unique(y_train)) == 2 else None

        name = f"model_{i+1}_{model_name}"
        garson_df = compute_garson_importance(pipeline, features_num,
name, fig_dir) if model_name == 'MLP' else pd.DataFrame()

        # Matriz de Confusão
        cm = confusion_matrix(y_test, y_pred)
        fig, ax = plt.subplots()
        sns.heatmap(cm, annot=True, fmt="d", cmap="Blues", ax=ax)
        ax.set_title(f'Matriz de Confusão — {name}')
        ax.set_xlabel('Predito')
        ax.set_ylabel('Real')
        plt.savefig(os.path.join(fig_dir,
f'{name}_confusion_matrix.png'))
        plt.close(fig)

        # Curva ROC
        if y_proba is not None:
            fpr, tpr, _ = roc_curve(y_test, y_proba)
            roc_auc = roc_auc_score(y_test, y_proba)
            fig, ax = plt.subplots()
            ax.plot(fpr, tpr, label=f'AUC = {roc_auc:.2f}')
            ax.plot([0, 1], [0, 1], linestyle='--')
            ax.set_title(f'Curva ROC — {name}')
            ax.set_xlabel('FPR')
            ax.set_ylabel('TPR')
            ax.legend()
            plt.savefig(os.path.join(fig_dir, f'{name}_roc_curve.png'))
            plt.close(fig)
        else:
            fpr, tpr, roc_auc = None, None, None

        final_report.append({
            'config_index': i,
            'model_name': model_name,
            'features': features,
            'params': row['params'],
            'roc_auc': roc_auc,
            'garson_weights': garson_df.to_dict('records') if not
garson_df.empty else None,
```

```
            'test_metrics': {
                'accuracy': (y_test == y_pred).mean(),
                'classification_report': classification_report(y_test,
y_pred, output_dict=True),
            },
            'confusion_matrix': cm.tolist(),
            'fpr_tpr': (fpr, tpr) if fpr is not None else None,
            'y_true': y_test.tolist(),
            'y_pred': y_pred.tolist(),
            'y_proba': y_proba.tolist() if y_proba is not None else
None
        })

    return pd.DataFrame(final_report)

# === MONTAGEM E SALVAMENTO NO DRIVE ===
def mount_drive():
    from google.colab import drive
    drive.mount('/drive')
    assert os.path.exists("/drive/My Drive/ArtigoLivia"), "Erro ao
montar o Google Drive!"

def save_to_drive(df, filename):
    base_dir = '/drive/My Drive/ArtigoLivia'
    os.makedirs(base_dir, exist_ok=True)
    path = os.path.join(base_dir, filename)
    df.to_excel(path, index=False)
    print(f"Salvo em: {path}")

# === EXECUÇÃO FINAL ===
mount_drive()

df_final_report = evaluate_top_models()
df_top5 = df_top5.reset_index().rename(columns={'index':
'config_index'})
df_final_report = df_top5.merge(df_final_report, on='config_index')

save_to_drive(df_final_report, 'ModelosTop5_Avaliacao_Completa.xlsx')
save_to_drive(df_top5, 'ResumoTop5_Optuna_Metricas.xlsx')
```
